# Long Intergenic Non-Protein Coding RNA 02381 Promotes the Proliferation and Invasion of Ovarian Endometrial Stromal Cells through the miR-27b-3p/CTNNB1 Axis

**DOI:** 10.3390/genes13030433

**Published:** 2022-02-26

**Authors:** Xiaoqing Wang, Peili Wu, Cheng Zeng, Jingwen Zhu, Yingfang Zhou, Ye Lu, Qing Xue

**Affiliations:** Department of Obstetrics and Gynecology, Peking University First Hospital, Beijing 100034, China; wxq0722@bjmu.edu.cn (X.W.); wupeili20090508@163.com (P.W.); vivian_0608@126.com (C.Z.); 2161008558@bjmu.edu.cn (J.Z.); zhouyf8853@163.com (Y.Z.); luye04988@bjmu.edu.cn (Y.L.)

**Keywords:** endometriosis, LINC02381, miR-27b-3p, CTNNB1, proliferation, invasion

## Abstract

Purpose: Catenin Beta 1 (CTNNB1) is a key regulator of cell proliferation and invasion in endometriosis; however, its upstream factor is not clear. Long noncoding RNAs may participate in endometriosis. The aim of this study was to investigate the mechanism of interaction between LINC02381 and CTNNB1 in endometriosis. Method: Screening and validation of RNAs were completed by whole transcriptional sequencing and qRT-PCR. The subcellular localization of LINC02381 was determined by RNA in situ hybridization and nucleo-cytoplasmic separation. Plasmids were transfected for functional experiments. Luciferase assay was used to verify the binding relationship. Results: The expression of LINC02381 and CTNNB1 was significantly increased in ovarian ectopic endometrial tissues (OSAs) and ectopic endometrial stromal cells (ESCs). When LINC02381 was downregulated in ESCs, the expression of CTNNB1, metallopeptidase 9 (MMP9) and cyclinD1, as well as ESCs invasion and proliferation, decreased. LINC02381 was mainly present in the cytoplasm of ESCs, indicating that it may act as a competitive endogenous RNA. Bioinformatic analysis revealed that microRNA-27b-3p (miR-27b-3p) is a downstream target of LINC02381. miR-27b-3p decreased in OSAs and ESCs. Moreover, when miR-27b-3p was upregulated in ESCs, the expression of CTNNB1, MMP9 and cyclinD1, as well as the invasion and proliferation ability of ESCs, were reduced. Additionally, rescue experiments demonstrated that the expression of CTNNB1, MMP9 and cyclinD1, as well as the invasion and proliferation ability, were significantly increased in the group transfected with both sh-LINC02381 and a miR-27b-3p inhibitor. Conclusion: LINC02381 upregulated CTNNB1 by adsorbing miR-27b-3p, causing increased proliferation and invasion of ESCs.

## 1. Introduction

Endometriosis is a benign gynecological disorder that affects 10% of reproductive-aged women. It is characterized by the outgrowth of endometrial cells from the lining of the uterus outside the uterine cavity [1], with pelvic pain and infertility being its main clinical manifestations [2,3,4]. The etiology of endometriosis is highly complex and diverse. Despite extensive investigation, the exact mechanism underlying endometriosis needs to be further clarified, and effective treatments are still required.

Sampson [5] first described endometriosis as being caused by retrograde movement of the endometrium through the fallopian tube, thereby invading and proliferating in tissues outside the uterus. To some extent, the proliferation and invasion of endometrial stromal cells (ESCs) are regulated by cyclinD1 and matrix metalloproteinase (MMP) 9, which are important markers of endometriosis [6,7]. In short, MMPs are capable of facilitating cell invasion in endometriosis by degrading the primary structure of the extracellular matrix and basement membrane [7]. In turn, cyclinD1, which is a cell cycle regulator, is essential for progression through the G1 phase and is a prominent prognostic marker for endometrial diseases, including endometriosis [7,8]. However, little is known about the upstream regulation of both cyclinD1 and MMP9.

CTNNB1, a key downstream molecule in the Wnt signal transduction pathway, has been a popular research topic in many fields [9,10], including liver disease, adrenal tumors, leukemia, endometrial cancer, stem cell self-renewal, and development and differentiation of reproductive tissue. In particular, researchers [11,12] believe that CTNNB1 can support endometriosis progression by promoting the proliferation and invasion of ESCs. In addition, cyclinD1 and MMP9 are downstream molecules of CTNNB1 [13,14]. Yue et al. [15] demonstrated that the Wnt/β-catenin pathway could regulate glioma cell proliferation and invasion, in part via the epidermal growth factor receptor signaling pathway that supports the transcription of cyclinD1 and MMP9. Furthermore, Matsuzaki et al. [16] confirmed that antibody-mediated interleukin-4 inhibition suppressed the development of endometriosis by blocking the expression of CTNNB1 and MMP9 in vivo. Thus, CTNNB1 is a key factor in the regulation of proliferation and invasion in endometriosis. However, few studies have focused on the upstream regulatory factors of CTNNB1.

Long noncoding RNAs (lncRNAs), which lack protein-coding capacity, represent more than 98% of the human transcriptome [17]. They are emerging as vital regulators in gene expression networks by controlling nuclear structure and transcription in the nucleus, as well as by regulating the stability, translation, and posttranslational modification of mRNAs in the cytoplasm [18]. Among their activities, the competitive endogenous RNA (ceRNA) mechanism is one of the important functions of lncRNAs, which is closely related to their cytoplasmic localization. Salmena et al. [19] proposed that ceRNA activity forms a large-scale regulatory network across the transcriptome, greatly expanding the functional genetic information in the human genome and playing important roles in pathological conditions. Therefore, several studies have evaluated the contributions of lncRNAs to various benign and malignant conditions to identify potential diagnostic markers [20,21]. In particular, microarray analyses of endometriosis cases have suggested that lncRNAs or ceRNAs are associated with the pathogenesis of endometriosis. However, few studies have conducted in-depth investigations of the underlying mechanisms, and the activity of ceRNAs in endometriosis remains unclear [8]. A newly discovered gene, LINC02381, was found to exacerbate rheumatoid arthritis by adsorbing the microRNA (miRNA) miR-590-5p [22] and to promote cell viability and migration by targeting miR-133b in cervical cancer cells [23]. Moreover, epigenetically silenced LINC02381 could function as a tumor suppressor by regulating the PI3K-Akt signaling pathway [24]. However, to date, no study has evaluated the relationship between LINC02381 and endometriosis. Based on the results of RNA fluorescence in situ hybridization (RNA FISH) and nuclear-cytoplasmic fractionation, we found that LINC02381 mainly existed in the cytoplasm of ESCs, suggesting that LINC02381 might play an important role as a ceRNA. Through bioinformatic and genetic analyses, we speculated that miR-27b-3p might be the downstream regulator of LINC02381 and upstream regulator of CTNNB1.

This study was performed to investigate the regulatory effect of LINC02381 on CTNNB1 and on ESC proliferation and invasion in endometriosis. We propose that LINC02381 could upregulate the expression of CTNNB1 by adsorbing miR-27b-3p in ESCs. In addition, the LINC02381/miR-27b-3p/CTNNB1 axis could promote the proliferation and invasion of ESCs, thereby accelerating the development of endometriosis.

## 2. Materials and Methods

### 2.1. Sample Selection and Primary Cell Culture

Samples of eutopic endometrium (EM) and ectopic endometrioma (OSA) from the cyst walls were obtained immediately from 21 patients, ages 23 to 40 years old, who underwent hysteroscopy combined with laparoscopy for endometriosis, forming nine pairs of self-control groups. The collected tissue samples were used for transcriptome analysis, total protein and RNA extraction, and primary cell culture. All women had regular menstrual cycles and were not taking hormone therapy. According to the preoperative history, the menstrual phase was confirmed, and all samples were in the proliferative phase. The tissue sample collection protocol was approved by the Peking University Institutional Review Board (No. 2020 (279)), and each patient signed an informed consent form to publish this paper before the samples were used.

Human endometrial stromal cells (EMs) and ESCs were isolated from the collected tissues as previously described [25]. Briefly, the tissue samples were washed 2–3 times with phosphate buffered saline (PBS) solution, cut into small pieces, and processed with collagenase (1 mg/mL) (Sigma, St. Louis, MO, USA) and DNase (0.04 mg/mL) (Sigma, St. Louis, MO, USA) for 1 h at 37 °C. Next, the mixture was filtered and centrifuged to remove tissue mass and epithelial cells. After centrifugation, the pellet was resuspended in the culture medium and placed in a 37 °C, 5% CO_2_ incubator. The medium was changed the next day to remove blood cells. The medium was composed of 10% fetal bovine serum (FBS, Cat.#PL16004; Gibco, Waltham, MA, USA), 89% Dulbecco’s modified Eagle’s medium (DMEM)/F12 (1:1, HyClone Laboratories, Logan, UT, USA), and 1% antibiotic antimycotic (Cat.#15240062, Gibco, Grand Island, NY, USA). 

### 2.2. RNA FISH

In order to ensure better permeability of the probe, the length of the probe was maintained within 200–400 bp. Two types of probes were designed to increase the signal intensity. The sequences were the following: LINC02381-a: forward 5’-CCTGATGGCCACTCACGC-3’ and reverse 5’-TAGATTTTCCAAAGTT-3’; LINC02381-b: forward 5’-TACTCAAAGCAATTCAAAATTCTCTCTTCT-3’ and reverse: 5’-GTACTAGAATATTAGGAATGAAAGCC-3’. Normal human genomic DNA was used as a template and the product was amplified by polymerase chain reaction (PCR). The product was then cloned into a plasmid using the T-A cloning method. The sequencing results proved that the products were correctly inserted. Using the PCR Fluorescein Labeling Mix (Cat.#11636154910) and Fluorescein-12-dUTP (Cat.#11373242910; Roche, Basel, Switzerland), the probe was labeled, purified, and quantified. Cells in the logarithmic growth phase were seeded on sterile slides. When the cells reached 70–80% confluence, the slides were removed from the culture and washed with PBS. Then, the slides were aged (56 °C for 30–60 s) and fixed with methanol and glacial acetic acid mixture (3:1) at room temperature for 20 min. Next, the slides were immersed for 2 min in 70%, 85%, and 100% ethanol solutions, and were then heated at 56 °C. The hybridized solution (containing 50% deionized formamide, (5×) SSC, (5×) Denhardt, 0.5% SDS, 100 μg/mL salmon sperm DNA and 10% dextran sulfate) was configured to ensure the probe concentration of 20–30 ng/μL (5–7 μL for each slide). They were placed at 73 °C for 5 min for denaturation and 50 °C for incubation reserve. Afterwards, the specimens were dripped into the hybridization solution, sealed, and denatured with glue. The next day, the edge banding and coverslips were removed. The slides were sequentially immersed in 50% deionized formamide, (1×) SSC at 42–48 °C for 15 min; (0.1×) SSC at 60 °C for 5 min thrice; followed by PBS plus 0.2% Tween-20 at 37 °C for 5 min thrice. The slides were then washed with PBS and sequentially submersed in 70%, 85%, and 95% ethanol solutions. After drying, the slides were placed in mounting medium with DAPI and incubated for confocal observation. At least five fields of view were analyzed from each sample.

### 2.3. Cytoplasmic and Nuclear RNA Purification

Cytoplasmic and nuclear separation and purification experiments were performed using the Cytoplasmic and Nuclear RNA Purification Kit (Cat#21000; Norgen Biotek, Thorold, Canada) according to the manufacturer’s instructions. Briefly, the cells in the logarithmic growth phase were collected and washed with PBS and placed on ice. Prepared Lysis Buffer J was added to the cells and, after shaking for 5 min, the cell lysate was transferred into an RNase-free tube and centrifuged tube at 14,000 rpm for 10 min. The supernatant containing the cytosolic RNA was transferred into a new RNase-free tube, whereas the nuclear RNA was left at the bottom of the original tube. Buffer SK and 100% ethanol (200 μL each) were successively added to the supernatant containing cytoplasmic RNA. The mixture was vortexed for 10 s and then centrifuged at 6000 rpm for 1 min. Next, 400 μL of Buffer SK and 200 μL of 100% ethanol were sequentially added to the pellet containing nuclear RNA and vortexed for 10 s, and centrifuged at 6000 rpm, for 1 min. After washing with Wash Solution A, the RNA samples were eluted with buffer E and stored at −80 °C.

### 2.4. Plasmid Construction and Transfection 

Plasmids encoding short hairpin RNA (shRNA) targeting *LINC02381* or scramble short hairpins (control) were constructed by Shanghai Genechem (Shanghai, China). At the beginning of the experiment, we constructed three kinds of shRNA plasmids and transfected them respectively, and we found that the effect of shRNA-LINC02381-1 (target sequence: GCCTCTGCTCTGTTCAGTT) was significantly better than that of shRNA-LINC02381-2 (target sequence: GCAGCTTGTTCCAGACCTT) and shRNA-LINC02381-3(target sequence: GCTGTTCATTCCACTAATA). Thus, shRNA-LINC02381-1 (expressed as sh-LINC02381) was widely used in subsequent experiments. Amplification and extraction of the plasmids were performed using the EndoFree Maxi Plasmid Kit (Cat#DP117; Tiangen Biotech, Beijing, China). The mimics (miR10000419), inhibitors (miR20000419), mimic-NC (miR1N0000001-1-5), and inhibitor-NC (miR2N0000001-1-5) of miR-27b-3p analogs and were constructed by Ribobio (Guangzhou, China). The transfection was performed using Lipofectamine 3000 Transfection Reagent (Cat#L3000015, Invitrogen, Waltham, MA, USA) according to the manufacturer’ s instructions.

### 2.5. Cell Counting Kit-8 (CCK8) Assay

The cells were collected when the culture reached 70–80% confluency, and were reseeded into 96-well plates, in six replicate wells at a density of 3 × 10^4^ cells/mL in 100 μL of medium. The CCK-8 reagent (10 µL; Dojindo Molecular Technologies, Xiongben, Japan) was added to each well and left to incubate for 1.5 h. The light absorption (OD) value was measured using a microplate reader at a wavelength of 450 nm. The time points of 0, 12, 24, 36, 48, 60, and 72 h were recorded and the cell proliferation curve was drawn based on the data measured.

### 2.6. Matrigel Invasion Assay

Invasion assays were performed using Matrigel-coated trans-wells (1:8 dilution; BD Company, Franklin Lakes, NJ, USA). The mixture was placed on the upper chamber surface and incubated at 37 °C for 30 min for Matrigel polymerization. Cells in the exponential growth phase were collected and 1 × 10^5^ cells were resuspended in serum-free medium and seeded in the upper chamber. Medium (600 μL) containing 20% FBS was added to the lower chamber. After 48 h of culture, the upper chambers were removed and washed thrice with PBS. The upper layer of redundant cells was removed with a cotton swab and the cells were fixed with 4% paraformaldehyde for 30 min. The cells were then dyed with 0.1% crystal violet for 30–60 min, and washed thrice with deionized water. At least five images were taken for each sample.

### 2.7. Western Blotting

Briefly, the cells were collected and resuspended in RIPA buffer containing protease and phosphatase inhibitors. The mixture was vigorously shaken on ice for 30 min and centrifuged. The supernatant containing the proteins was collected and the total protein concentration in each sample was determined using the BCA method (KeyGen Biotech, Nanjing, China) at a wavelength of 562 nm. For the Western blot, 15 µg of protein per well were loaded into the gel. After the electrophoresis (80 V for 30 min, 120 V for 1 h) and transference (200 mA for 2 h), the membrane containing the proteins was blocked with 5% milk blocking solution for 1 h. The membrane was incubated overnight with anti-β-catenin (1:1000; Cat#ab32572), anti-MMP9 (1:1000, Cat#ab76003), and anti-cyclin D1 (1:1000; Cat#ab76003) from Abcam (Cambridge, UK), and anti-β-tubulin (1:1000; Cat#2128T) from Cell Signaling Technology (Danvers, MA, USA). The next day, the membranes were washed thrice with tris-buffered saline solution with Tween-20, and incubated with a horseradish enzyme labeled goat anti-rabbit secondary antibody (1:1000; Cat#ZB-2301) from ZSGB-BIO (Beijing, China). ImageJ (U.S. National Institutes of Health, Bethesda, MD, USA) was used to evaluate the proteins and perform densitometry analysis of the target proteins using β-tubulin as control. 

### 2.8. Real-Time Quantitative PCR

Total RNA was extracted using Trizol (Invitrogen) and was reverse transcribed using the TranScript miRNA First-Strand cDNA Synthesis SuperMix (Cat#AT351-01) and the TranScript One-Step gDNA Removal and cDNA Synthesis SuperMix (Cat#AT311-03) from TransGen Biotech (Beijing, China), according to the manufacturer’s instructions. The target genes (LINC02381, CTNNB1, miR-27b-3p, MMP9, and CCND1) and the internal reference gene (GAPDH, ACTB, U1, U6) were amplified by qRT-PCR using the ABI Power SYBR Green gene expression system (Applied Biosystems, Waltham, MA, USA) and the TranScript miRNA First-Strand cDNA Synthesis SuperMix (TransGen Biotech). The relative expression of the target gene was obtained by the 2^−ΔΔCT^ method. The primers used were the following: LINC02381(LOC400043) forward5’-CGGAGCAGAACACCCCTGATT-3’, reverse5’-CTGGAACAAGCTGCCTTCCTT-3’; CTNNB1forward5’ GGCTATTGTAGAAGCTGGTGGA-3’, reverse5’-TCTGAACAAGACGTTGACTTGGA-3’; GAPDH forward5’-GAAGGTGAAGGTCGGAGTC-3’, reverse5’-GAAGATGGTGATGGGATTTC-3’; β-actin forward 5’-CACGGCTGCTTCCAGCTC-3’, reverse 5’-CACAGGACTCCATGCCCAG-3’; MMP9 forward 5’-AGACCTGGGCAGATTCCAAAC-3’, reverse 5’-CGGCAAGTCTTCCGAGTAGT-3’; cyclinD1 forward 5’-CAATGACCCCGCACGATTTC-3’, reverse5’-CATGGAGGGCGGATTGGAA-3’; miR-27b-3p forward 5’-TTCACAGTGGCTAAGTTCTGC-3’ reverse(universal primer in transcript miRNA first strand cDNA synthesis Supermix (AT351-01) ), U1 forward 5’-ACTTACCTGGCAGGGGAGAT-3’, reverse 5’-TGCAGTCGAGTTTCCCACAT-3’; U6 forward 5’-CTCGCTTCGGCAGCACA-3’, reverse5’-AACGCTTCACGAATTTGCGT-3’.

### 2.9. Dual-Luciferase Reporter Gene Assay

The Dual-Luciferase Reporter Gene Assay was carried out by Ribobio (Guangzhou, China). In brief, 293T cells in the logarithmic growth phase were transplanted into 96-well plates and transfected with plasmids of mimic-miR-27b-3p, mimic-NC, LINC02381(NR_026656.1 complete sequence)-WT or CTNNB1(NM_001904.4 complete sequence)-WT. The luciferase reaction was performed using the Dual GLO^®^ Luciferase Assay System (Promega, USA). The reporter fluorescence of the vector used was the Rinilla luciferase gene (hrluc), and the corrected fluorescence was the firefly luciferase gene (hluc) (internal reference correction). The total length of LINC02381 and CTNNB1 were cloned downstream of the hrluc gene. The miR-27b-3p acted on the target gene (LINC02381 and CTNNB1) through the 3’ UTR region. Therefore, the miRNA was co-transferred with the constructed reporter gene vector, The interaction between miRNA and target gene was confirmed by the down-regulation of the relative fluorescence value of reporter gene.

### 2.10. Whole-Transcriptome Sequencing

Five pairs of OSA and EM samples were sent for whole-transcriptome sequencing (performed by Novogene, Beijing, China). In short, first, to analyze the differentially expressed lncRNAs and mRNAs in the two groups, the Ballgown suite includes functions for interactive exploration of the transcriptome assembly, visualization of transcript structures and feature-specific abundances for each locus, and post hoc annotation of assembled features to annotated features. Transcripts with a P-adjust <0.05 or Pval < 0.05 were assigned as differentially expressed. Second, in order to analyze the differentially expressed miRNAs, differential expression analysis of two groups was performed using the DESeq R package (1.8.3). The *P*-values was adjusted using the Benjamini and Hochberg method. The corrected *P*-value of 0.05 was set as the threshold for significantly differential expression by default. Third, Miranda was used to predict the targeting relationship between miRNA and genes, and the DESeq R package (1.8.3) was used to draw the interaction network diagram. The research method was provided by Novogene, Beijing, China.

### 2.11. Statistical Analyses

All data were analyzed using IBM SPSS Statistics for Mac, Version 22.0 (IBM Corp., Armonk, NY, USA). Two groups were compared using independent sample *t*-tests, and one-way analysis of variance (ANOVA) was used to compare three or more groups. In our experiment, a total of 5 pairs of self-control tissue samples were selected for whole transcriptome sequencing. In the qRT-PCR experiment, the differential expression of LINC02381 (21 pairs of tissues and 16 pairs of cells), miR-27b-3p (15 pairs of tissues and 12 pairs of cells) and CTNNB1 (15 pairs of tissues and 13 pairs of cells) were repeatedly tested. In CCK-8, Transwell, nucleocytoplasmic separation, RNA-FISH and Western blot experiments, there are at least three samples in each group. Differences were considered statistically significant at *P* < 0.05. 

## 3. Results

### 3.1. The lncRNA/miRNA/mRNA Interaction Network

**Figure 1:**
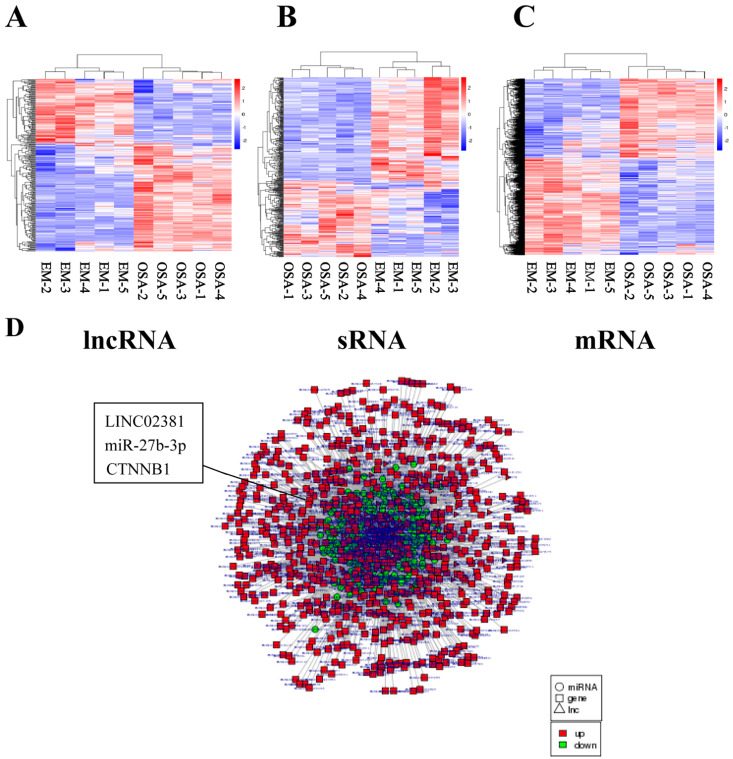
The lncRNA/miRNA/mRNA interaction network. Five pairs of ectopic endometrial tissue (OSA) and eutopic endometrial tissue (EM) samples from women with endometriosis were sent for whole-transcriptome sequencing. sRNA: small RNA. (**A**–**C**) Heatmap of the differentially expressed lncRNAs, sRNAs, and mRNAs. (**D**) The lncRNA/miRNA/mRNA regulatory interactions were formed according to the bioinformatics analysis.

According to the results of previous studies, ceRNA is a widespread mechanism that regulates gene expression in many biological contexts, including endometriosis [8]. LncRNAs and mRNAs can compete to bind to miRNAs and thus regulate each other. A lncRNA–miRNA–mRNA gene interference network in OSA and EM was designed, which provided a broad range of information for the search for ceRNA regulatory mechanisms. Five pairs of OSA and EM samples from women with endometriosis were sent for whole-transcriptome sequencing (performed by Novogene, Beijing, China). The collected data revealed 217, 3234, and 402 differentially expressed lncRNAs, mRNAs, and miRNAs, respectively. The related heatmap is shown in Figure 1A–C. According to the bioinformatics analysis (Figure 1D), 96,409 lncRNA/miRNA/mRNA regulatory interactions were detected, among which 80,167 regulatory interactions were in the up-down-up mode and 16,242 regulatory interactions were in the down-up-down mode. The identified network included interactions between miRNAs and RNAs, lncRNAs and miRNAs, as well as lncRNAs–RNAs interactions.

### 3.2. Expression and Distribution of LINC02381

**Figure 2:**
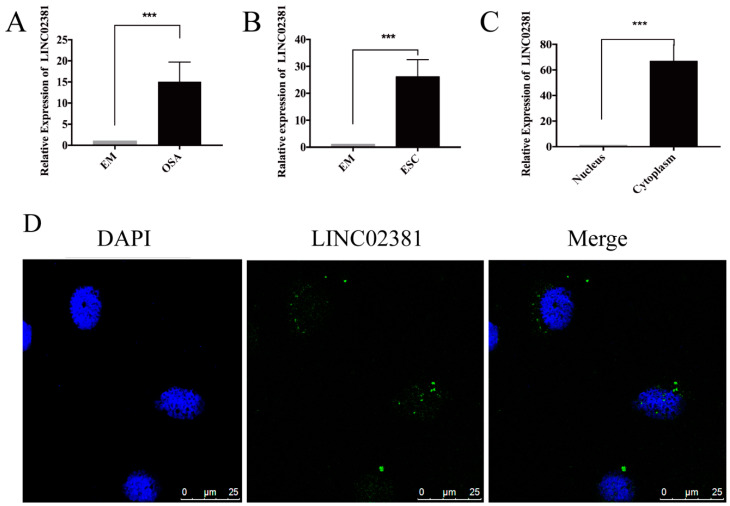
Expression and distribution of LINC02381. (**A**) Paired OSA and EM samples were harvested for real-time quantitative polymerase chain reaction (qRT-PCR) to quantify the RNA expression of LINC02381 (*n* = 21, *P* < 0.001). (**B**) Paired ectopic endometrial stromal cells (ESCs) and eutopic endometrial stromal cells (EMs) were collected for qRT-PCR to quantify the RNA expression of LINC02381 (*n* = 17, *P* < 0.001). (**C**) ESCs were harvested for cytoplasmic and nuclear fractionation experiments and qRT-PCR (*n* = 3, *P* = 0.000). (**D**) ESCs were harvested for RNA fluorescent in situ hybridization (FISH) to determine the distribution of LINC02381. *** *P* < 0.001 vs. individual controls.

Based on the data from the whole-transcriptome sequencing, we found that LINC02381, which is a relatively new lncRNA, was significantly upregulated in OSA compared with EM samples. The qRT-PCR results further confirmed that the expression of LINC02381 in OSA was 14.84 times higher than that in EM (*n* = 21, *P* < 0.001, Figure 2A). Similarly, the expression of LINC02381 in ESCs was 26.16 times higher than that in EMs (*n* = 16, *P* < 0.001, Figure 2B). It is well known that lncRNA activity is closely related to its subcellular localization [26]. Therefore, to clarify the subcellular distribution of LINC02381, nuclear and cytoplasmic fractionation extracts from ESCs were collected and subjected to qRT-PCR. The results showed that LINC02381 expression was 66.80 times higher in the cytoplasm than in the nucleus (*n* = 3, *P* = 0.000, Figure 2C). Accordingly, the FISH results showed that LINC02381 (Figure 2D) was mainly distributed in the cytoplasm of ESCs rather than the nucleus. Thus, LINC02381 may play a main biological role in the cytoplasm of ESCs.

### 3.3. LINC02381 Enhances CTNNB1 Expression

**Figure 3:**
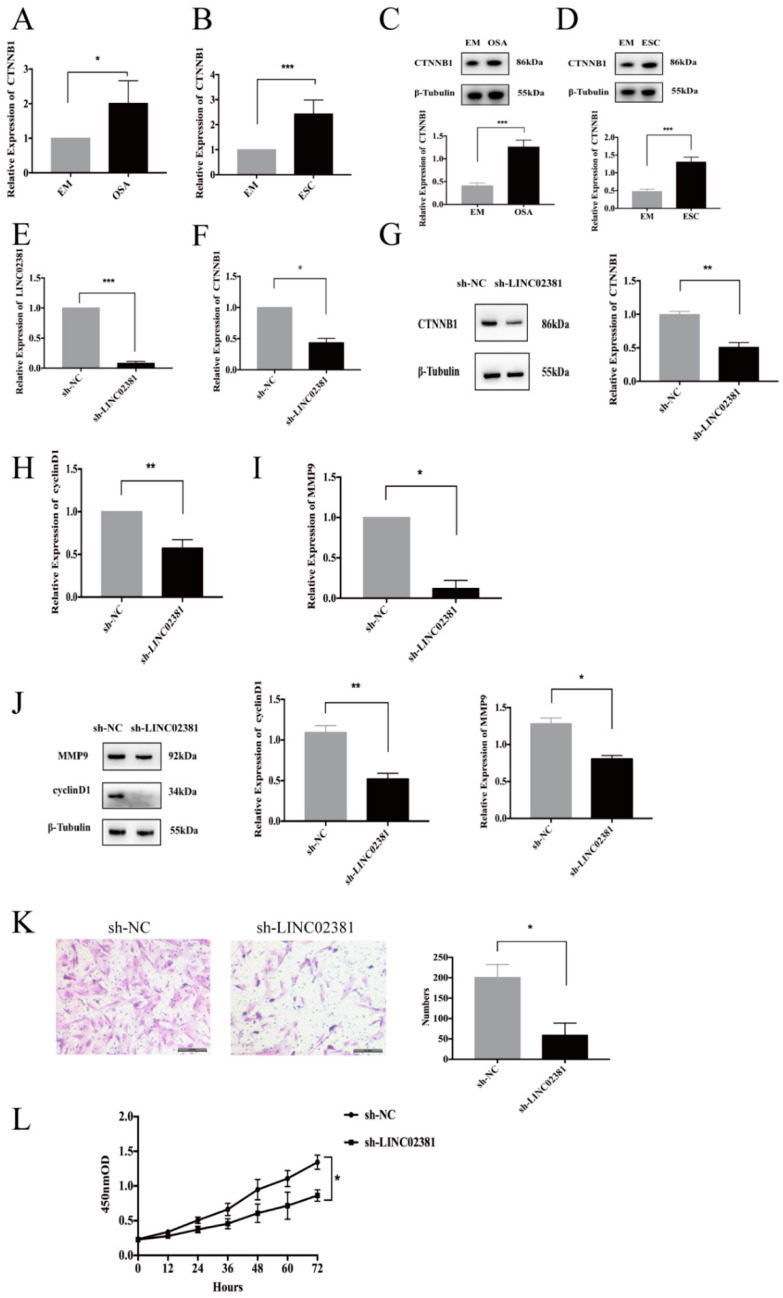
LINC02381 enhances CTNNB1 expression and the proliferation and invasion of ESCs. (**A**) Paired OSA and EM samples were collected for qRT-PCR to quantify the mRNA expression of CTNNB1 (*n* = 15, *P* < 0.05). (**B**) Paired ESCs and EMs were collected for qRT-PCR to quantify the mRNA expression of CTNNB1 (*n* = 13, *P* < 0.001). (**C**) Paired OSA and EM samples were collected for Western blotting to determine the protein expression of CTNNB1 (*n* = 9, *P* < 0.001). (**D**) Paired ESCs and EMs were collected for Western blotting to determine the protein expression of CTNNB1 (*n* = 5, *P* < 0.001). (**E**,**F**) ESCs were cultured for qRT-PCR after LINC02381 knockdown to quantify the RNA expression of LINC02381 (*n* = 3, *P* < 0.001) and CTNNB1 (*n* = 3, *P* < 0.05). (**G**) ESCs were cultured for Western blotting after LINC02381 knockdown to test the protein expression of CTNNB1 (*n* = 3, *P* < 0.01); (**H**,**I**) ESCs were cultured for qRT-PCR to quantify the mRNA expression of cyclinD1 (*n* = 3, *P* < 0.01) and MMP9 (*n* = 3, *P* < 0.05) when LINC02381 was knocked down. (**J**) ESCs were cultured for Western blotting to detect the protein expression of cyclinD1 (*n* = 3, *P* < 0.05) and MMP9 (*n* = 3, *P* < 0.01) following the decrease in LINC02381. (**K**) ESCs were cultured for Transwell assays after LINC02381 was knocked down (*n* = 3, *P* < 0.05). (**L**) ESCs were cultured for CCK-8 assays after LINC02381 was knocked down (from 24 h to 72 h, *n* = 3, *P* < 0.05). * *P* < 0.05, ** *P* < 0.01, *** *P* < 0.001.

The above ceRNA networks indicated that LINC02381 could modulate the expression of CTNNB1 in OSA. Thus, the expression of CTNNB1 was evaluated by qRT-PCR and Western blot assays in paired tissues and cells. At the mRNA level, CTNNB1 expression was 2.01 times higher in OSA than in EM (*n* = 15, *P* < 0.05, Figure 3A), and it was 2.43 times higher in ESCs than in EMs (*n* = 13, *P* < 0.001, Figure 3B). Additionally, CTNNB1 protein levels were significantly elevated in both OSA (*n* = 9, *P* < 0.001, Figure 3C) and ESCs (*n* = 5, *P* < 0.001, Figure 3D).

To elucidate the potential molecular mechanisms through which LINC02381 contributes to the progression of endometriosis, sh-LINC02381 and control plasmids were transfected into ESCs. As shown by qRT-PCR, CTNNB1 expression decreased by 57% (*n* = 3, *P* < 0.05, Figure 3F) after 98.4% knockdown of LINC02381 (*n* = 3, *P* = 0.000, Figure 3E). Western blot analysis also showed that when LINC02381 was knocked down, CTNNB1 levels decreased by 49.2% (*n* = 3, *P* < 0.01, Figure 3G). In summary, LINC02381 could regulate CTNNB1 expression in ESCs. 

### 3.4. LINC02381 Promotes the Proliferation and Invasion of ESCs

Proliferation and invasion have been considered downstream events of CTNNB1. To verify the role of LINC02381 in proliferation and invasion in endometriosis, the expression of cyclinD1 and MMP9 upon LINC02381 silencing in ESCs was evaluated. qRT-PCR results showed that when LINC02381 was reduced, the expression of both cyclinD1 and MMP9 was decreased by 42.7% (*n* = 3, *P* < 0.01, Figure 3H) and 87.99% (*n* = 3, *P* < 0.05, Figure 3I), respectively, at the mRNA level and by 37.25% and 52.38% (*n* = 3, *P* < 0.05 and *P* < 0.01, Figure 3J), respectively, at the protein level. Moreover, when LINC02381 was knocked down, the invasion potential of ESCs was significantly reduced (sh-NC: 200.85 ± 17.50, sh-LINC02381: 59.13 ± 6.97; *n* = 3, *P* < 0.05, Figure 3K). The proliferation of ESCs showed a similar trend, being significantly reduced upon LINC02381 knockdown (from 24 to 72 h, *n* = 3, *P* < 0.05, Figure 3L). Altogether, these findings revealed a novel function of LINC02381 in regulating the proliferation and invasion of ESCs, most likely by controlling cyclinD1 and MMP9 expression.

### 3.5. The LINC02381/miR-27b-3p/CTNNB1 Regulatory Axis 

**Figure 4:**
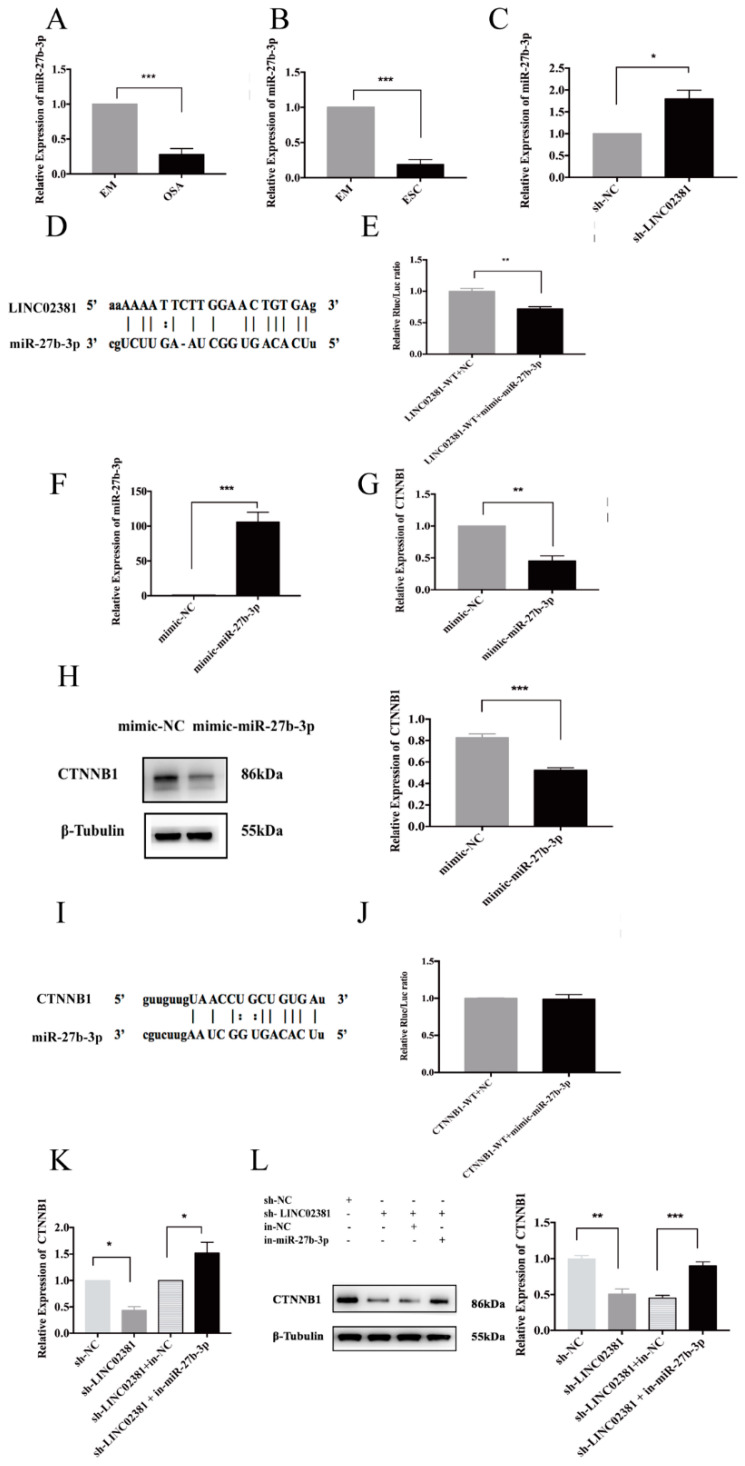
The LINC02381/miR-27b-3p/CTNNB1 regulatory axis. (**A**,**B**) Paired OSA and EM samples and paired ESCs and EMs were collected for qRT-PCR to quantify the RNA expression of miR-27b-3p (*n* = 15, *P* < 0.001; *n* = 12, *P* < 0.001, separately). (**C**) ESCs were harvested for qRT-PCR after LINC02381 knockdown (*n* = 3, *P* < 0.05). (**D**) The miRanda software was used to predict the binding relationship between LINC02381 and miR-27b-3p. (**E**) 293T cells were collected for Dual-Luciferase Reporter Gene Assay after transfection with indicated plasmids (*n* = 3, *P* < 0.01). (**F**,**G**) ESCs were collected for qRT-PCR after transfection with mimic-miR-27b-3p and mimic-NC. The efficiency of miR-27b-3p overexpression (*n* = 3, *P* < 0.001); the mRNA expression of CTNNB1 decreased after overexpression of miR-27b-3p (*n* = 3, *P* < 0.01). (**H**) The protein expression of CTNNB1 in ESCs decreased after miR-27b-3p overexpression (*n* = 3, *P* < 0.001). (**I**) The miRanda software was used to predict the binding relationship between CTNNB1 and miR-27b-3p. (**J**) 293T cells were collected for Dual-Luciferase Reporter Gene Assay after transfection with indicated plasmids (*n* = 3, *P* > 0.05). (**K**) ESCs were collected for qRT-PCR to quantify the mRNA expression of CTNNB1 after transfection with the indicated plasmids (*n* = 3, *P* < 0.05, both). (**L**) ESCs were cultured for Western blotting to determine the protein expression of CTNNB1 after transfection with the indicated plasmids (*n* = 3, *P* < 0.01; *n* = 3, *P* < 0.001, separately); * *P* < 0.05, ** *P* < 0.01, *** *P* < 0.001.

Since cytoplasmic-localized lncRNAs can act as ceRNAs to regulate miRNAs, additional experiments were performed to identify the downstream miRNAs of LINC02381 based on the ceRNA network generated from the whole-transcriptome sequencing analysis. The data showed that miR-27b-3p expression in OSA samples was 71.99% lower than that in EM samples (*n* = 15, *P* < 0.001, Figure 4A). Consistent with these findings, the expression of miR-27b-3p in ESCs was 81.12% lower than that in EMs (*n* = 12, *P* < 0.001, Figure 4B). Additionally, when LINC02381 expression in ESCs was knocked down by 98.4% (*n* = 3, *P* = 0.000, Figure 3E), the expression of miR-27b-3p increased 1.8-fold (*n* = 3, *P* < 0.05, Figure 4C). The miRanda software was used to predict the binding relationship between LINC02381 and miR-27b-3p (Figure 4D). Dual-Luciferase Reporter Gene Assay (Figure 4E) showed that the relative Luciferase activity of 293T cells was significantly decreased after co-transfection of mimic-miR-27b-3p and LINC02381-WT (*n* = 3, *P* < 0.01). Therefore, these results indicated that miR-27b-3p could be a direct downstream target of LINC02381.

To ascertain the regulatory relationship between miR-27b-3p and CTNNB1, miR-27b-3p was overexpressed in ESCs, and CTNNB1 expression was analyzed. Compared with that in the mimic-NC group, the expression of miR-27b-3p was increased by 105-fold in the miR-27b-3p mimics group (*n* = 3, *P* = 0.000, Figure 4F), whereas CTNNB1 was reduced by 55% (*n* = 3, *P* < 0.01, Figure 4G). Protein analysis by Western blotting further confirmed that CTNNB1 levels were reduced by 37% (*n* = 3, *P* = 0.000, Figure 4H) following the overexpression of miR-27b-3p. Bioinformatics software was used to predict the targeting relationship between miR-27b-3p and CTNNB1 (Figure 4I). In Figure 4J, Dual-Luciferase Reporter Gene Assay indicated that there was no direct binding relationship between miR-27b-3p and CTNNB1.

Interestingly, when ESCs were co-transfected with both the sh-LINC02381 plasmid and a miR-27b-3p inhibitor, CTNNB1 expression increased 1.52-fold (*P* < 0.05) compared with that in cells transfected with sh-LINC02381 alone, which showed 57% lower CTNNB1 expression (*n* = 3, *P* < 0.05, Figure 4K). Similarly, the protein expression of CTNNB1 decreased by 49.2% (*P* < 0.01) when LINC02381 was knocked down alone and increased 1.99-fold in the rescue experiment (*n* = 3, *P* = 0.000, Figure 4L).

### 3.6. The LINC02381/miR-27b-3p/CTNNB1 Regulatory Axis Is Involved in ESCs Proliferation and Invasion 

**Figure 5:**
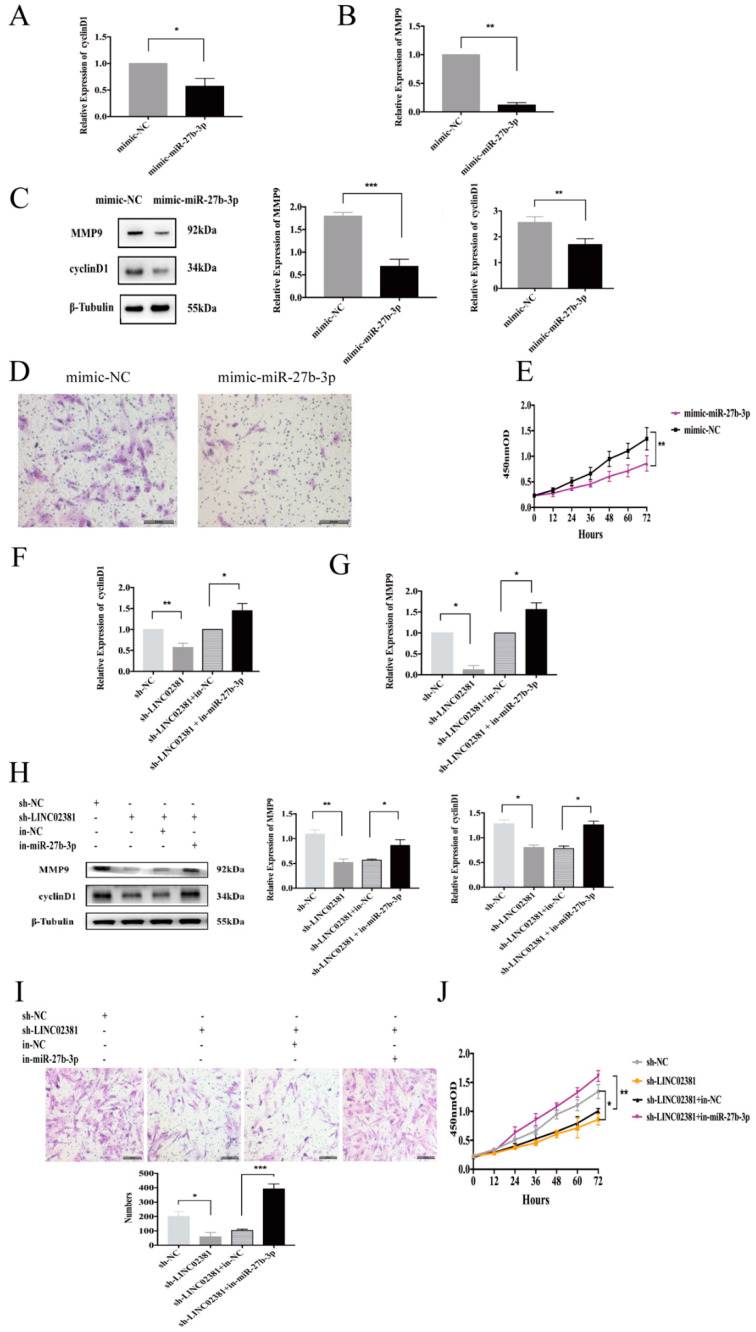
The LINC02381/miR-27b-3p/CTNNB1 regulatory axis. (**A**,**B**) ESCs were harvested for qRT-PCR after miR-27b-3p expression was increased to quantify the mRNA expression of cyclinD1 (*n* = 3, P < 0.05) and MMP9 (*n* = 3, *P* < 0.01). (**C**) ESCs were harvested for Western blot analysis after mimic-miR-27b-3p/mimic-NC was added to determine the protein expression of cyclinD1 (*n* = 3, *P* < 0.01) and MMP9 (*n* = 3, *P* < 0.001). (**D**) ESCs were harvested for Transwell assays after the expression of miR-27b-3p increased to detect the invasive ability (*n* = 3, *P* < 0.001). (**E**) ESCs were harvested for CCK-8 assays after the expression of miR-27b-3p increased to detect the proliferative ability (*n* = 3, *P* < 0.01). (**F**,**G**) ESCs were harvested for qRT-PCR after the indicated plasmids were added to quantify the mRNA expression of cyclinD1 (*n* = 3, *P* < 0.01; *n* = 3, *P* < 0.05, separately) and MMP9 (*n* = 3, *P* < 0.05, both). (**H**) Cells were collected for Western blotting after transfection with the indicated plasmids. LINC02381 knockdown reduced cyclinD1 levels by 37.25% (*n* = 3, *P* < 0.05) and MMP9 levels by 52.38% (*n* = 3, *P* < 0.01). After the rescue experiment, cyclinD1 increased 1.61-fold (*n* = 3, *P* < 0.05) and MMP9 1.52-fold (*n* = 3, *P* < 0.05). (**I**) The indicated plasmids were transfected into ESCs, and cell invasion was evaluated (*n* = 3, *P* < 0.05; *n* = 3, *P* < 0.001, separately). (**J**) ESCs were harvested and subjected to CCK-8 assays after transfection (from 24 to 72 h, *n* = 3, *P* < 0.05; *n* = 3, *P* < 0.01, separately). * *P* < 0.05, ** *P* < 0.01, *** *P* < 0.001.

To further verify that the LINC02381/miR-27b-3p/CTNNB1 regulatory axis is involved in proliferation and invasion in endometriosis and to clarify the role of LINC02381 in endometriosis, we designed the following experiments. First, miR-27b-3p mimics were transfected into ESCs to elucidate the function of miR-27b-3p on the biological behavior of ESCs. The expression of cyclinD1 and MMP9 was reduced by 42.73% and 88.90% (*n* = 3, *P* < 0.05 and *P* < 0.01, Figure 5A,B), while the protein levels of cyclinD1 and MMP9 were reduced by 33.53% and 61.90% (*n* = 3, *P* < 0.01 and *P* = 0.000, Figure 5C). With the increasing expression of miR-27b-3p, the invasion potential of ESCs was markedly reduced (mimic-NC: 375.67 ± 20.60, mimic-miR-27b-3p: 69.33 ± 6.51; *n* = 3, *P* = 0.000, Figure 5D), as was their proliferative capacity (from 24 to 72 h, *n* = 3, *P* < 0.01, Figure 5E). Therefore, miR-27b-3p could reduce the proliferation and invasion of ESCs.

Rescue experiments further confirmed the effect of this axis on the proliferation and invasion of ESCs. The mRNA expression of cyclinD1 and MMP9 was increased 1.45-fold (*n* = 3, *P* < 0.05) and 1.56-fold in ESCs transfected with both the sh-LINC02381 plasmid and the miR-27b-3p inhibitor (*n* = 3, *P* < 0.05, Figure 5F,G). Furthermore, the protein levels of cyclinD1 and MMP9 showed an identical trend (Figure 5H), with cyclinD1 and MMP9 being increased 1.61-fold (*n* = 3, *P* < 0.05) and 1.52-fold (*n* = 3, *P* < 0.05) in the above co-transfected ESCs. Of note, when both miR-27b-3p and LINC02381 were blocked, the invasive ability of ESCs significantly increased (sh-LINC02381+in-NC: 103.00 ± 8.89, sh-LINC02381+in-miR-27b-3p: 392.33 ± 33.50, *n* = 3, *P* = 0.000, Figure 5I). However, no significant difference was observed between the sh-LINC02381 group and sh-LINC02381+in-NC group. Moreover, the proliferation of ESCs also significantly increased when both miR-27b-3p and LINC02381 were blocked (from 24 to 72 h, *n* = 3, *P* < 0.01, Figure 5J).

Taken together, these findings indicate that LINC02381 may promote the expression of cyclinD1 and MMP9 and induce the proliferation and invasion of ESCs by repressing miR-27b-3p.

## 4. Discussion

In this study, it was verified that the expression of LINC02381 is elevated in ectopic endometrial tissues and cells [8]. Knockdown of LINC02381 released the expression of miR-27b-3p, leading to the reduction in ESCs proliferation. On the other hand, simultaneous inhibition of miR-27b-3p and LINC02381 induced the expression of CTNNB1, which in turn, enhanced the proliferation of ESCs, suggesting that the LINC02381/miR-27b-3p/CTNNB1 axis is a potential prognostic biomarker and therapeutic target for endometriosis.

It is widely recognized that ceRNA regulatory networks exist in patients with endometriosis. Upon further screening and verification of the differentially expressed lncRNAs by whole-transcriptome sequencing and qRT-PCR, LINC02381 was herein identified as a potentially relevant regulatory factor in endometriosis. Similar to our findings, LINC02381 has been reported to participate in rheumatoid arthritis and cervical cancer [22,23]. However, to our knowledge, LINC02381 is still a relatively novel lncRNA that has not been studied in detail in other pathological conditions. This is the first report demonstrating that LINC02381 is highly expressed in both OSAs and ESCs compared with eutopic tissues and cells. Thus, it is reasonable to hypothesize that LINC02381 may be related to the occurrence of endometriosis. The results of FISH and nuclear-cytoplasmic fractionation experiments consistently showed that LINC02381 was mainly distributed in the cytoplasm rather than in the nucleus of ESCs. Therefore, LINC02381 may be active in the cytoplasm, thereby being involved in ceRNA mechanisms [18] and potentially functioning as an endogenous miRNA sponge. LINC02381 was described to target miR-133b in cervical cancer cells [23], and it could exacerbate rheumatoid arthritis by adsorbing miR-590-5p and activating the mitogen-activated protein kinase signaling pathway in fibroblast-like synoviocytes [22]. In light of the ceRNA network provided by our whole-transcriptome sequencing analysis, it was possible to screen potential downstream targets of LINC02381, among which the LINC02381/miR-27b-3p/CTNNB1 regulatory axis was identified in OSA and ESCs of patients with endometriosis.

Previous studies have reported an association between miR-27b-3p and endometriosis. Ferlita and colleagues indicated that two isoforms of the miR-27 family (miR-27a and miR-27b) seemed to be involved in different endometrial lesions [27]. In addition, Kim et al. proposed that ginsenoside Rg3 effectively altered the fibrous properties of ESCs from patients with endometriosis, which was closely associated with miR-27b-3p modulation [28]. This further validated our finding that miR-27b-3p plays an important role in the development of endometriosis. However, the specific mechanism underlying the association between miR-27b-3p and endometriosis has not been reported. Importantly, our data confirmed for the first time, the targeting relationship between miR-27b-3p and LINC02381. Furthermore, when miR-27b-3p was overexpressed, the expression of CTNNB1 decreased, which confirmed our previous hypothesis. In agreement with these findings, the relationship between CTNNB1 and endometriosis has been reported in several studies [29,30,31,32], which is related to the Wnt and the ERK1/2 pathways [33,34]. To the best of our knowledge, CTNNB1 [11] plays an important role in the adhesion, invasion, and metastasis of endometrial tissues via the Wnt/β-catenin signaling pathway and could even improve the border of extraovarian endometriosis lesions [35]. Moreover, researchers [36,37] have suggested that CTNNB1 regulation could lead to distortion in the mesoderm, which in turn would promote the abnormal localization of stem cells and induce the occurrence of endometriosis [36,37]. Thus, CTNNB1 may be involved in the pathogenesis of endometriosis by participating in the mesenchymal-to-epithelial transition and epithelial-to-mesenchymal transition processes. Altogether, these findings further confirmed the role of CTNNB1 in the proliferation and invasion of ESCs. According to previous reports, CTNNB1 may be involved in multiple ceRNA regulatory axes [38,39,40,41], such as the SNHG11/miR-4436a/CTNNB1 ceRNA axis [38] and the DSCR8/miR-485-5p/CTNNB1 axis [40]. Herein, new upstream regulatory factors of CTNNB1 were identified, namely, LINC02381 and miR-27b-3p, as demonstrated by several experiments showing that when LINC02381 decreased, miR-27b-3p significantly increased, whereas CTNNB1 decreased. The results of the Dual-Luciferase Reporter Gene Assay did not indicate the binding relationship between miR-27b-3p and CTNNB1, we believed that an indirect relationship might exist between miR-27b-3p and CTNNB1. Furthermore, cyclinD1 and MMP9 were not indicated to be modulated by LINC02381 or miR-27b-3p in our ceRNA network or public database. Therefore, we believe that these two genes were probably not directly downstream of LINC02381 and miR-27b-3p. Based on previous studies [13,14], cyclinD1 and MMP9 were likely downstream of CTNNB1. Moreover, CTNNB1 might translocate into the nucleus in order to activate cyclinD1, MMP9, and other genes. Before that, CTNNB1 accumulates in the cytoplasm. LINC02381 might act like a scaffold and somehow help the accumulation of CTNNB1 in the cytoplasm and protect it from phosphorylation and ubiquitin degradation. However, the specific relationship between them remains to be confirmed by further experiments.

Our study is a preliminary exploration. Nonetheless, the limitation of the current study lies in the lack of relevant animal experiments for further verification. The future challenge relies on a better understanding of why such regulatory networks exist in patients with endometriosis, how they may evolve, and how to use these regulatory networks in clinical applications. People [14] believe that finding reliable biomarkers is imperative for disease diagnosis, a concept that has been widely accepted. In addition, the combination of multiple markers is known to have better diagnostic performance than a single marker by improving the sensitivity and reducing both the false positive and negative rates. Nevertheless, the LINC02381/miR-27b-3p/CTNNB1 regulatory axis identified in this study may offer insights into the diversity and complexity of lncRNA/mRNA interactions and contribute to the application of such biomarkers in the future diagnosis of endometriosis.

## 5. Conclusions

In summary, LINC02381 may be able to upregulate CTNNB1 by adsorbing miR-27b-3p, causing increased migration and invasion of ESCs, thus leading to the occurrence and development of endometriosis.

## Data Availability

The data that support the findings of this study are available from the corresponding author upon reasonable request.
